# Meta-analysis of the radiological and clinical features of Usual Interstitial Pneumonia (UIP) and Nonspecific Interstitial Pneumonia (NSIP)

**DOI:** 10.1371/journal.pone.0226084

**Published:** 2020-01-13

**Authors:** Lukas Ebner, Stergios Christodoulidis, Thomai Stathopoulou, Thomas Geiser, Odile Stalder, Andreas Limacher, Johannes T. Heverhagen, Stavroula G. Mougiakakou, Andreas Christe

**Affiliations:** 1 Department of Diagnostic, Interventional and Pediatric Radiology, Inselspital, Bern University Hospital, University of Bern, Switzerland; 2 ARTORG Center for Biomedical Engineering Research, University of Bern, Switzerland; 3 Department for Pulmonary Medicine, Inselspital, Bern University Hospital, University of Bern, Switzerland; 4 CTU Bern and Institute of Social and Preventive Medicine (ISPM), University of Bern, Switzerland; Toranomon Hospital, JAPAN

## Abstract

**Purpose:**

To conduct a meta-analysis to determine specific computed tomography (CT) patterns and clinical features that discriminate between nonspecific interstitial pneumonia (NSIP) and usual interstitial pneumonia (UIP).

**Materials and methods:**

The PubMed/Medline and Embase databases were searched for studies describing the radiological patterns of UIP and NSIP in chest CT images. Only studies involving histologically confirmed diagnoses and a consensus diagnosis by an interstitial lung disease (ILD) board were included in this analysis. The radiological patterns and patient demographics were extracted from suitable articles. We used random-effects meta-analysis by DerSimonian & Laird and calculated pooled odds ratios for binary data and pooled mean differences for continuous data.

**Results:**

Of the 794 search results, 33 articles describing 2,318 patients met the inclusion criteria. Twelve of these studies included both NSIP (338 patients) and UIP (447 patients). NSIP-patients were significantly younger (NSIP: median age 54.8 years, UIP: 59.7 years; mean difference (MD) -4.4; p = 0.001; 95% CI: -6.97 to -1.77), less often male (NSIP: median 52.8%, UIP: 73.6%; pooled odds ratio (OR) 0.32; p<0.001; 95% CI: 0.17 to 0.60), and less often smokers (NSIP: median 55.1%, UIP: 73.9%; OR 0.42; p = 0.005; 95% CI: 0.23 to 0.77) than patients with UIP. The CT findings from patients with NSIP revealed significantly lower levels of the honeycombing pattern (NSIP: median 28.9%, UIP: 73.4%; OR 0.07; p<0.001; 95% CI: 0.02 to 0.30) with less peripheral predominance (NSIP: median 41.8%, UIP: 83.3%; OR 0.21; p<0.001; 95% CI: 0.11 to 0.38) and more subpleural sparing (NSIP: median 40.7%, UIP: 4.3%; OR 16.3; p = 0.005; 95% CI: 2.28 to 117).

**Conclusion:**

Honeycombing with a peripheral predominance was significantly associated with a diagnosis of UIP. The NSIP pattern showed more subpleural sparing. The UIP pattern was predominantly observed in elderly males with a history of smoking, whereas NSIP occurred in a younger patient population.

## Introduction

Idiopathic pulmonary fibrosis (IPF) constitutes the most prevalent type of idiopathic interstitial pneumonia (IIP), accounting for 55% of IIP cases [1]. IPF is known to occur in adult individuals aged greater than 50 years and affects more men than women [1–3]. In addition, IPF is thought to be associated with cigarette smoking, as many patients with IPF are former or current smokers [1–3]. The prevalence of IPF in the USA is reported to be 63 cases per 100,000 population and up to 23.4 cases per 100,000 population in Europe. The incidence of IPF in the USA ranges from 6.8 to 17.4 per 100,000 population and 0.22–7.4 per 100,000 population in Europe [4]. The median survival time reported in recent studies ranges from 2 to 5 years, starting at the time of diagnosis; this survival time is worse than in patients with many types of cancer [5]. IPF is associated with the radiographic and pathological patterns known as usual interstitial pneumonia (UIP). The UIP pattern can be associated with several other entities, such as rheumatoid arthritis, certain medications or chronic hypersensitivity pneumonitis.

Nonspecific interstitial pneumonia (NSIP), on the other hand, represents a pathological subtype of IIP that can mimic IPF in its clinical presentation and has a more favorable prognosis, with a median survival time of more than 9 years. NSIP accounts for 25% of IIP cases, constituting the second most common type of IIP after IPF [3]. NSIP shows a slight female predominance and typically occurs in a younger patient population than IPF [6]. Similar to patients with UIP, the secondary NSIP pattern on a computed tomography (CT) scan can also be linked to collagen vascular disease and other entities among the spectrum of autoimmune diseases.

The diagnosis of IIP requires background clinical information. Several studies have shown significant inter- and intraobserver variability in the radiological diagnosis of IIPs of up to 50% [7], which affect the overall diagnostic accuracy.

The main CT features of IPF are reported to be basal and peripheral reticulations, which are most typically associated with honeycombing potentially predicting patient outcomes [8–10]. Ground-glass opacities (GGOs) are also common, but less extensive. For NSIP, the reported characteristic CT patterns overlap with those of UIP and consist of GGOs and/or reticular patterns, while honeycombing is rare. However, chronic NSIP might develop into a fibrotic form termed fibrosing NSIP. When typical UIP patterns are present, an IPF diagnosis is made based on high-resolution CT (HRCT) images. In these cases, histopathological confirmation may not be required, according to recent guidelines. However, if HRCT findings are equivocal, a biopsy is still necessary [11]. Overall, the diagnostic accuracy of HRCT for UIP and NSIP has been reported to be up to 70% [12].

By achieving a reliable diagnosis based on imaging features, patients potentially avoid the risks of bleeding and general anesthesia and the high costs associated with a surgical biopsy [13–16]. Although UIP and NSIP imaging features have been described extensively in the literature and were incorporated in the diagnostic guidelines, no systematic review of the literature has been conducted. Our aims were to review the literature and summarize the most pertinent findings for UIP and NSIP and to provide an evidence-based approach.

The goal of this systematic review was to provide an overview of the prevalence and location of CT patterns and typical medical variables (age, sex, and smoking status) for UIP and NSIP. We sought to determine the patterns and variables that best discriminated between UIP and NSIP. For exact numbers, suitable studies were pooled into this review.

## Materials and methods

The reporting of the results from this systematic review was organized according to Preferred Reporting Items for Systematic Reviews and Meta-Analysis (PRISMA) guidelines [17].

### Eligibility criteria

The following criteria were applied to select the studies: (1) a dedicated research article (no letters or abstracts were considered); (2) adequate imaging studies (HRCT) including volume scans or HRCT sequences with a slice thickness of less than 2 mm; (3) a detailed description of radiological NSIP and/or UIP patterns on CT images according to the established guidelines [2]; and (4) a confirmed diagnosis of UIP or NSIP based on biopsy specimens or a board decision (dedicated ILD board composed of specialized pneumologists, radiologists and pathologists in a tertiary care setting, as recommended by the American Thoracic Society (ATS), European Respiratory Society (ERS), and Fleischner Society [12,18,19]).The following exclusion criteria were applied: (1) case reports and (2) studies of less than 10 cases. Additionally, review articles and studies with insufficient subject identification were excluded from the analysis. In the case of redundant reporting of patient populations, we only included the study with the largest sample size. We considered only studies published in English and French. All suitable studies were stored in a portable document format and transferred to Papers software (ReadCube, The Netherlands). The titles and abstracts of all manuscripts were screened by one author (A.C., who has 18 years of experience in chest radiology). Manuscripts were then separately analyzed for eligibility by one author (A.C.). A second validation of the preselected articles was conducted by a different author (L.E., who has 5 years of expertise in chest imaging). Duplicates were removed from the article list.

### Information sources and search strategy

We performed a literature search of the PubMed/Medline and Embase databases. We applied the following search terms to titles and abstracts: "Lung Diseases, Interstitial/diagnosis", "Lung Diseases, Interstitial/diagnostic imaging", "Pulmonary Fibrosis/diagnosis", "Pulmonary Fibrosis/diagnostic imaging" and pattern, reticula*, honeycombing, ground-glass, peribronchovascular, bronchovascular, traction bronchiectasis, tractionbronchiectasis, UIP [Title/Abstract] or NSIP, and radiography and fibro* [Title] OR idiopathic* [Title], pneumonia* [Title])) and (("1992/01/01" [PDat]: "3000/12/31" [PDat]) NOT case report* [tiab]) NOT (therap* [Title] OR treat* [Title]). Detailed information on the Embase search strategy can be retrieved from Appendix 1. We searched for articles published between 1992 and 2017. The complete literature search was conducted in May 2017. The rationale for the search dates was owed to the extensive amount of data that has been screened by the clinical radiologists as well as validated by experts. Further statistical analysis was time consuming as well. However, to the best of our knowledge, no substantial contributions to the existing data were published since then. Also, we are not aware of any similar meta-analysis that has been published on this topic to date.

### Study selection and data collection processes

Data extraction and coding were performed by one investigator (A.C.). All data were collected in a standardized worksheet (Microsoft Excel). This analysis included studies deemed relevant, including randomized controlled trials, cohort studies, and cross-sectional studies. The extracted parameters included patient demographics and smoking history (current smokers, former smokers and never smokers). The radiological patterns denoted by CT included honeycombing, GGOs, consolidation, and reticulation. In addition, the dominant pattern was recorded if mentioned in the manuscript. The extent of these patterns was noted by the percent (%) of the total lung volume. Studies indicating a radiologist’s estimate were pooled with studies mentioning only a per-lobe analysis (middle lobe or lingula counted as 1/4 of the right or left lung, and the lower lobe counted as 1/2 of the lung). The pattern distribution was recorded axially (inner 2/3 of the lungs versus the outer 1/3; the peribronchial distribution was considered the inner 2/3 of the lungs) and along the z-axis (upper or lower lobe predominance (below the level of the carina) or diffuse lung involvement). Data regarding involvement below the level of the carina were pooled with data regarding involvement of the lower lobe, middle lobe or lingula.

### Risk of bias in individual studies

The quality of the included studies in this systematic review was assessed with Quality Assessment of Studies of Diagnostic Accuracy included in Systematic Reviews (QUADAS) [20].

### Data extraction, quality assessment and statistical analysis

We compared study characteristics among UIP and NSIP studies. The number of complete observations for each arm and the median, minimum and maximum were summarized. The Wilcoxon test was applied to identify significant differences. For the 12 studies with both arms (UIP and NSIP), the number of complete observations and the median, minimum and maximum for each arm are displayed. We used random-effects meta-analysis described by DerSimonian & Laird and calculated pooled odds ratios (ORs) for binary data and pooled mean differences (MDs) for continuous data, together with 95% confidence intervals and p-values using the Stata command metan. Due to the low number of included studies, we also used the method by Paule-Mandel to assess robustness of pooled estimates using the Stata command admetan. The Stata function metan was used to estimate the pooled effect considering a random effect model using the DerSimonian-Laird’s method. The Stata function admetan was used to estimate the pooled effect considering a random effect model, using the Paule-Mandel’s method. The Wilcoxon–Mann–Whitney test was applied to compare the results of all arm studies with two-arm studies.

Heterogeneity was quantified using the I^2^ measure [21, 22]. An I^2^ larger than 50% denotes moderate heterogeneity, and a value larger than 75% indicates severe heterogeneity. The effect measures of the individual studies, the pooled measures, and the I^2^ measure and its p-value are shown in plots. The dashed red vertical line depicts the overall pooled OR or MD. The width of the diamond represents the 95% CI. All analyses were performed using Stata 14 software (Stata Corporation, College Station, Texas).

## Results

### Study selection

**Fig 1:**
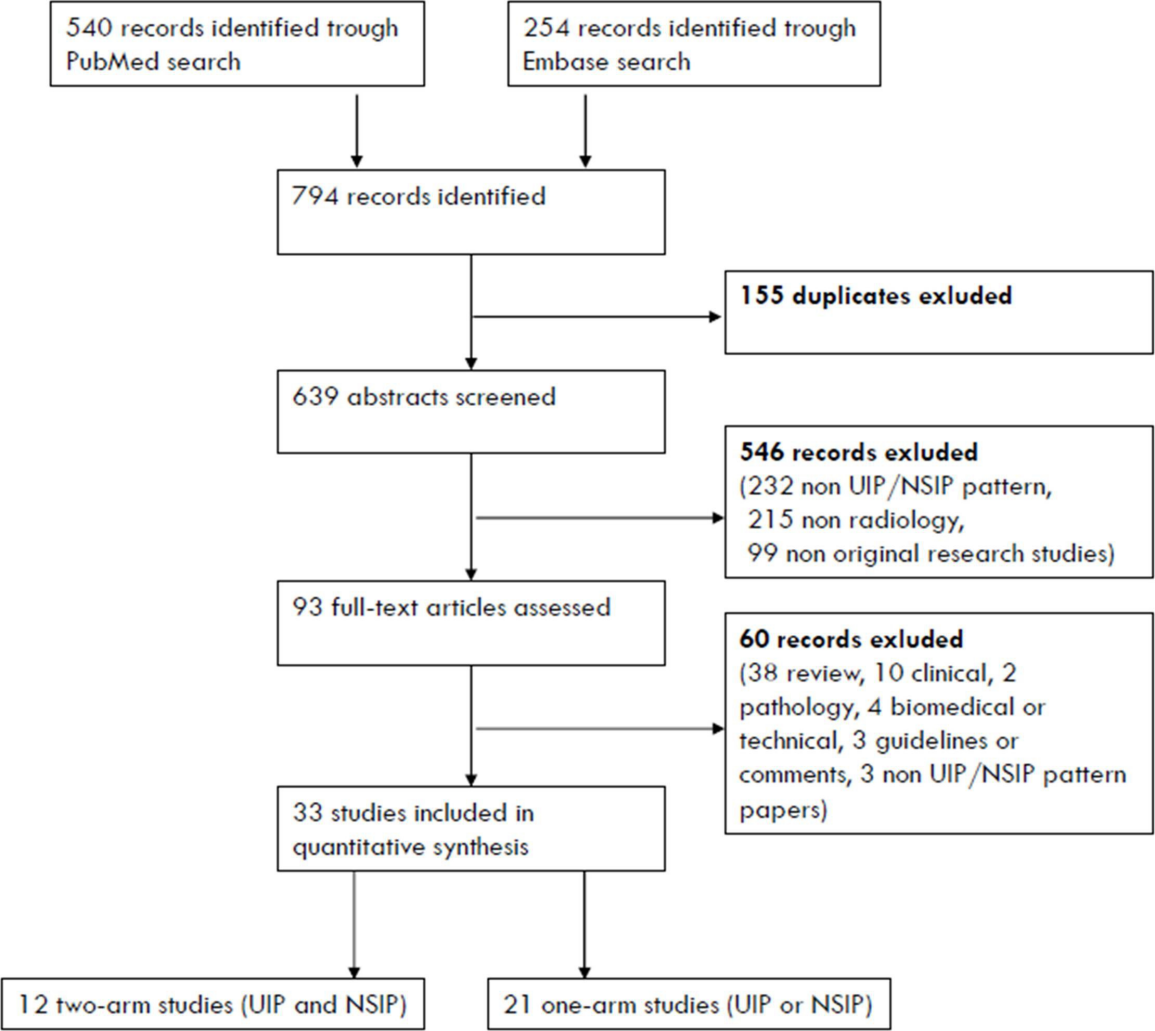
Preferred reporting items for systematic reviews and meta-analyses (PRISMA) flowchart showing the selection process.

After screening 639 abstracts, only 33 articles met the inclusion criteria. Of these 33 articles, only 12 studies were two-arm studies that simultaneously analyzed the prevalence of CT patterns in patients with NSIP and UIP [23–34]. A flow diagram was generated for the inclusion and exclusion criteria according to the PRISMA guidelines (Fig 1), and the characteristics of the included two-arm studies are listed in Table 1.

**Table 1:** Characteristics of the included two-arm studies.

Study Nr	Study (author)	Year	Ref.	ILD	Biopsy-proven	ILD board approved	Idiopathic forms (secondary cause excluded)	Sample size	Age (mean)	age (mean) standard deviation	Male (n)	Smoking (ever and past smokers; n)	Total disease extent (mean % of Lung volume)	GGO (y/n)	GGO extent (mean % of Lung volume)	Honeycombing (y/n)	Honeycombing extent (mean % of Lung volume)	Consolidation (y/n)	Consolidation extent (mean % of Lung volume)	Reticulation (y/n)	Reticulation extent (mean % of Lung volume)	Predominant GGO: GGO>(R+H)	Predominat reticulation: (R+H)>GGO	H: H and/or R > G	Subpleural sparing (y/n)	Emphysema (y/n)	Emphysema extent (mean % of Lung volume)	Mosaic GGO (y/n)	Peripheral predominance (outer 1/3, n)	Central predominance/perbronchial (inner 2/3, n)	Diffuse axial distribution (n)	Upper lung predominant distribution (n)	Lower lung predominant (below carina, n)	Diffuse distribution: both upper and lower lungs (n)
1	Kondoh et al	2005	[23]	NSIP	1		1	12	55.3	6.6	4	4																						
				UIP	1		1	27	56.0	10.9	20	20																						
2	Sumikawa et al	2014	[24]	NSIP	1		1	39	57.7	10.7	24	22	35.9		19.8		0.2		5.7						12		1.8		17	16	6			
				UIP	1	1	1	75	62.9	8.2	55	46	39.0		18.4		1.5		4.4						7		1.7		59	11	5			
3	Aaløkken et al	2012	[25]	NSIP	1	1	1	28					22.9		5.6		12.5		1.6		7.3													
				UIP	1	1	1	36					24.5		1.4		30.3		1.1		6.8													
4	Sumikawa et al	2012	[26]	NSIP	1	1	1	10						5		1		4		1									4	5	1		8	
				UIP	1	1	1	25						8		14		4		12									22	2	1		21	
5	Akira	2009	[27]	NSIP	1		1	54	59.2	9.5		29	15.8		8.3		0.4		2.7			8	25	0	22	3	7.3			20				
				UIP	1		1	42	61.4	8.7		31	17.8		5.6		3.0		1.1			2	39	6	0	7	9.7			2				
6	Silva et al	2008	[28]	NSIP	1			25	54.1	12.9	6		38.8	25		8		1		25					16			9	18		7	0	23	2
				UIP	1		1	23	62.0	6.9	17		22.6	23		16		0		23					1			10	18		5	0	19	4
7	Sumikawa et al	2006	[29]	NSIP	1		1	32							25.5		0.5		10.5								2.0		12			0	30	2
				UIP	1		1	20							20.0		4.0		6.0								6.0		15			0	20	0
8	Tsubamoto et al	2005	[30]	NSIP	1	1		36							31.3		1.7		10.8										12	22	2	1	32	3
				UIP	1	1		11							17.6		21.0		3.3										10	1	0	0	11	0
9	Jeong et al	2005	[31]	NSIP	1			25					33.0		20.0		2.0		3.0		9.0													
				UIP	1			70					35.0		18.0		7.5		0.5		9.5													
10	Elliot et al	2005	[32]	NSIP	1			25	50.0	12.0						8								1					19	0	6			
				UIP	1		1	22	58.0	11.0						17								9					21	0	1			
11	MacDonald et al	2001	[33]	NSIP	1		1	21	54.3		12		37.1		17.6				0.0			6	6.25											
				UIP	1		1	32	53.1		22		44.0		11.7				0.0			2.75	18.25											
12	Nagai et al	1998	[34]	NSIP	1		1	31			15	18		26		8		23																
				UIP	1		1	64			55	53		0		63		0																

### Risk of bias within studies

**Fig 2:**
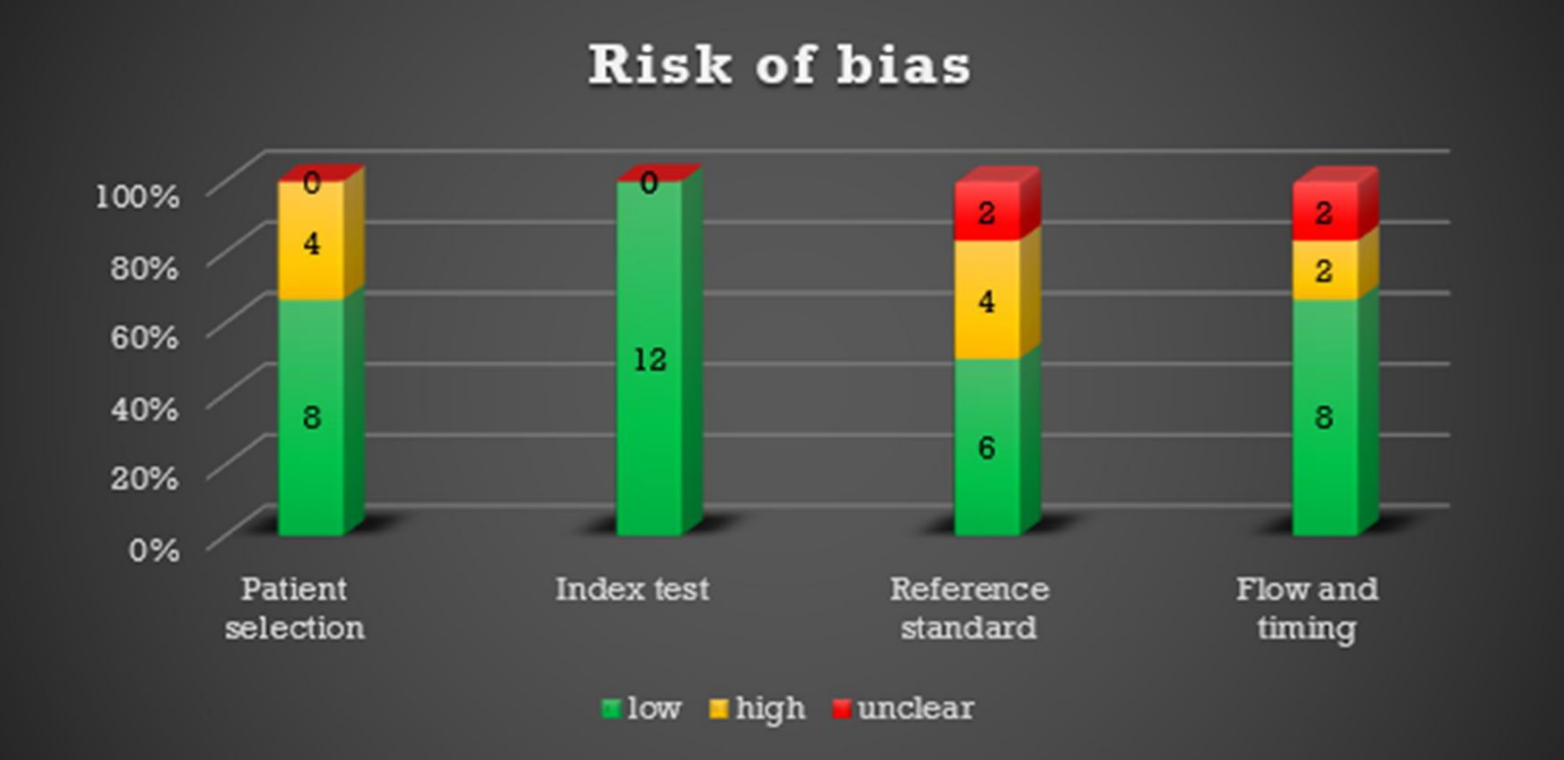
QUADAS-2: Number of studies with a low, high or unclear risk of bias.

**Fig 3:**
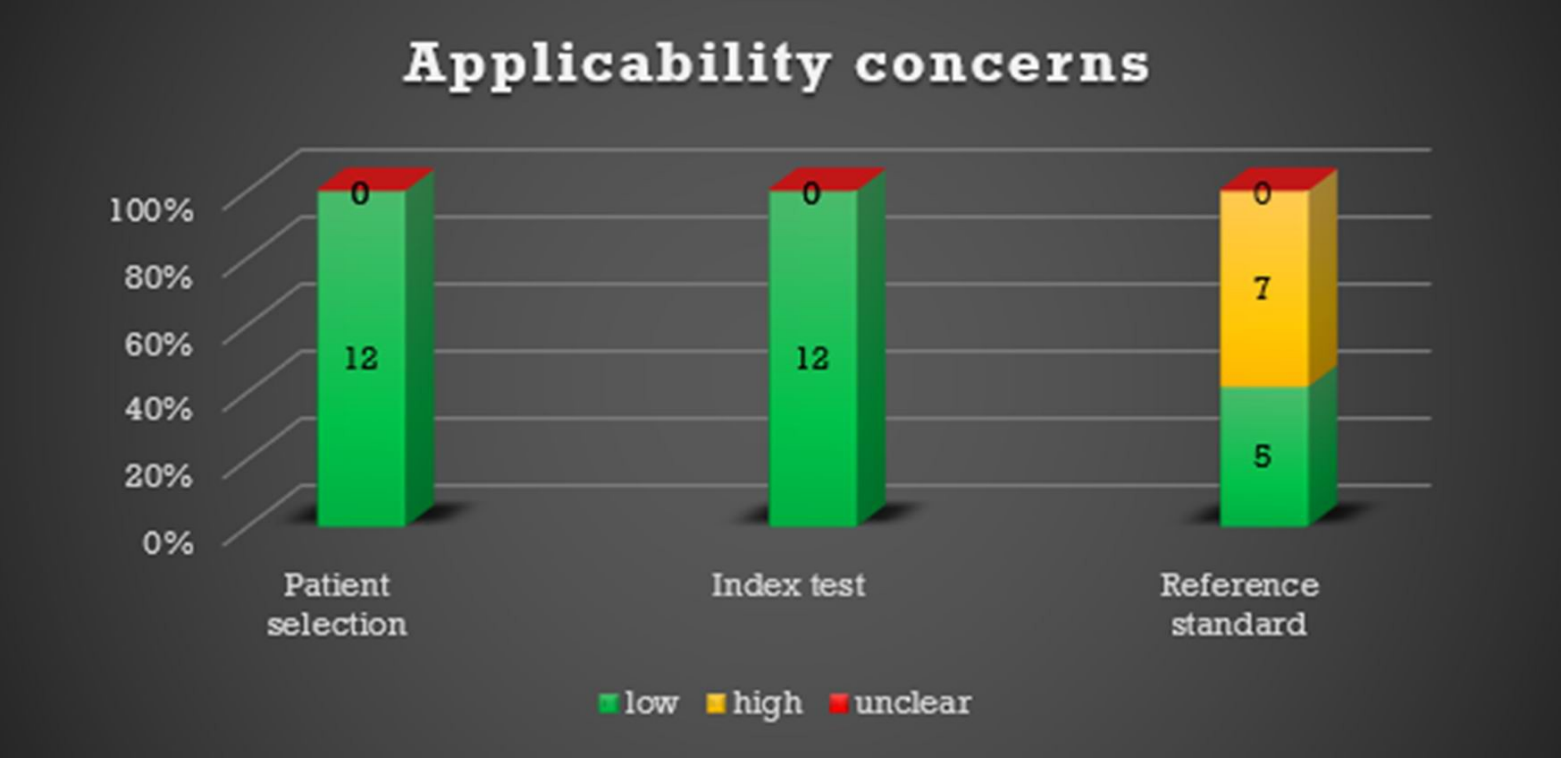
QUADAS-2: Number of studies with low, high or unclear concerns regarding applicability.

Due to the small number of studies, we performed a sensitivity analysis using the method of Paule-Mandel to assess robustness of pooled estimates, which yielded very similar results (data shown in appendix 2). A comparison of patterns between studies with only idiopathic cases and idiopathic and secondary UIP or NSIP cases did not show any significant differences (all p>0.1). Likewise, significant differences were not observed between biopsy-proven and ILD board-proven studies (all p>0.1). Therefore, the pooling of these subgroups is likely free of bias. The QUADAS-2 results are shown in Figs 2 and 3.

### Study characteristics

Thirty-three studies were selected according to the inclusion criteria, including 725 patients with NSIP and 1,593 patients with UIP. Twelve studies included both patients with UIP (447 patients) and NSIP (338 patients), eight studies included only patients with NSIP, and 13 studies included only patients with UIP. In 85% (17/20) of the NSIP study arms, the diagnosis was confirmed by a biopsy. In 30% (6/20) of the NSIP study arms, patients were diagnosed by a multidisciplinary ILD board of lung specialists (pulmonologists, pathologists and radiologists). In 50% (10/20) of the NSIP study arms, the final diagnosis of idiopathic NSIP was reached by excluding any other secondary etiologies. In 72% (18/25) of the UIP study arms, the diagnosis was confirmed by biopsy and histology (possible or incompatible UIP pattern on CT). In 48% (12/25) of the UIP study arms, patients were diagnosed by the dedicated ILD board. In 80% (20/25) of the UIP study arms, the diagnoses were classified as idiopathic without an identifiable etiology.

### Patient history and the prevalence, distribution and extent of UIP and NSIP CT patterns (Tables 2, 3 and 4)

The characteristics and statistics of all study arms (20 NSIP studies+25 UIP studies) and two-arm studies (12 NSIP+12 UIP) are listed in Tables 2 and 3. No significant differences in the median observations were observed between the two groups (p>0.2 for each variable). In the 12 studies that included patients with UIP and NSIP, patients with NSIP were significantly younger (NSIP: median age 54.8 years, UIP: 59.7 years; MD -4.4; p = 0.001), less often male (NSIP: median 52.8%, UIP: 73.6%; OR 0.32; p<0.001), and less often smokers than patients with UIP (NSIP: median 55.1%, UIP: 73.9%; OR 0.42; p = 0.005). Among patients with NSIP, significantly fewer cases with the honeycombing pattern (NSIP: median 28.9%, UIP: 73.4%; OR 0.07; p<0.001) and less peripheral disease predominance (NSIP: median 41.8%, UIP: 83.3%; OR 0.21 p<0.001) were observed. GGOs (NSIP: median 83.9%, UIP: 32%; OR 32.0; p = 0.256) and subpleural sparing (NSIP: median 40.7%, UIP = 4.3%; OR 16.3; p = 0.005) were more prevalent in patients with NSIP than in patients with UIP. Reticulations occurred in patients with UIP and NSIP, but the difference was not significant (NSIP: median 55.0%, UIP: 74.0%; OR 0.12; p = 0.06).

**Table 2:** Patient history, CT patterns, distribution and extent of NSIP and UIP (one- and two-arm studies).

	NSIP	UIP	Wilcoxon test
	n observations, median (min; max)	n observations, median (min; max)	p-value
Number of patients	725	1593	
Total number of studies (n)	n = 20	n = 25	
Age	n = 14, 54.6 (48.0; 59.2)	n = 19, 62.7 (53.1; 75.5)	<0.001
Male (%)	n = 14, 34.7 (4.3; 64.0)	n = 18, 73.6 (50.8; 86.1)	<0.001
Smoking (ever and past smokers) (%)	n = 9, 50.0 (8.2; 58.1)	n = 14, 73.2 (24.2; 91.7)	0.001
TOT disease extent (mean % of lung volume)	n = 9, 27.9 (0.4; 37.1)	n = 9, 32.0 (0.2; 44.0)	0.566
GGO (y/n) (%)	n = 8, 91.9 (44.3; 100.0)	n = 6, 31.0 (0.0; 100.0)	0.065
GGO extent (mean % of lung volume)	n = 12, 19.9 (5.6; 31.8)	n = 10, 14.7 (1.4; 23.4)	0.086
Honeycombing (y/n) (%)	n = 9, 8.5 (0.0; 32.0)	n = 10, 66.7 (15.9; 98.4)	0.001
Honeycombing extent (mean % of lung volume)	n = 10, 0.4 (0.0; 12.5)	n = 10, 5.6 (1.0; 30.3)	0.006
Consolidation y/n (%)	n = 8, 37.4 (4.0; 74.6)	n = 7, 2.9 (0.0; 58.8)	0.049
Consolidation extent (mean % of lung volume)	n = 11, 3.0 (0.0; 10.8)	n = 10, 1.1 (0.0; 6.0)	0.09
Reticulation (y/n) (%)	n = 7, 86.9 (10.0; 100.0)	n = 5, 100.0 (46.2; 100.0)	0.449
Reticulation extent (mean % of lung volume)	n = 7, 7.3 (3.3; 11.8)	n = 5, 10.8 (6.8; 30.1)	0.123
Predominant GGO: GGO>(R+H) (%)	n = 2, 21.7 (14.8; 28.6)	n = 3, 8.7 (4.8; 9.4)	0.083
Predominant reticulation: (R+H)>GGO (%)	n = 2, 37.4 (28.6; 46.3)	n = 2, 74.6 (56.3; 92.9)	0.121
H and/or R > G (%)	n = 2, 2.0 (0.0; 4.0)	n = 2, 27.6 (14.3; 40.9)	0.121
Subpleural sparing (y/n) (%)	n = 5, 30.8 (21.3; 64.0)	n = 3, 4.3 (0.0; 9.3)	0.025
Emphysema (y/n) (%)	n = 2, 11.7 (11.5; 11.9)	n = 4, 19.3 (0.0; 32.3)	0.355
Emphysema extent (mean % of lung volume)	n = 5, 1.8 (1.0; 3.2)	n = 5, 2.8 (1.6; 6.8)	0.251
Mosaic GGO (y/n) (%)	n = 1, 36.0 (36.0; 36.0)	n = 4, 16.4 (8.0; 43.5)	0.48
Peripheral predominance (outer 1/3) (%)	n = 8, 44.7 (33.3; 76.0)	n = 8, 82.5 (75.0; 95.5)	0.001
Central predominance/perbronchial (inner 2/3) (%)	n = 7, 37.0 (0.0; 61.1)	n = 7, 5.0 (4.1; 14.7)	0.064
Diffuse axial distribution both (%)	n = 7, 27.1 (5.6; 47.5)	n = 7, 6.7 (0.0; 21.7)	0.035
Upper lung predominant distribution (%)	n = 5, 0.0 (0.0; 2.8)	n = 5, 1.0 (0.0; 8.3)	0.435
Lower lung predominant (below carina) (%)	n = 5, 91.8 (80.0; 96.0)	n = 5, 84.0 (78.6; 100.0)	0.917
Diffuse distribution: both upper and lower lungs (%)	n = 5, 8.2 (6.3; 11.9)	n = 5, 16.2 (0.0; 20.4)	0.6

GGO: ground-glass opacity, H: honeycombing; R: reticulation; y/n: yes/no

**Table 3:** Patient demographics, CT patterns, distribution and extent of NSIP and UIP.

	NSIP	UIP	Pooled OR	95%-CI	p-value	I-squared (%)
Binary data (y/n)	n observations, median (min; max)	n observations, median (min; max)				
Male (%)	n = 6, 52.8 (24.0; 64.0)	n = 6, 73.6 (68.8; 85.9)	0.32	(0.17 to 0.60)	<0.001	48.78
Smoking (ever and past smokers) (%)	n = 4, 55.1 (33.3; 58.1)	n = 4, 73.9 (61.3; 82.8)	0.42	(0.23 to 0.77)	0.005	35.40
GGO (y/n) (%)	n = 3, 83.9 (50.0; 100.0)	n = 3, 32.0 (0.0; 100.0)	*32.049	(0.081 to 12655)	0.256	92.44
Honeycombing (y/n) (%)	n = 4, 28.9 (10.0; 32.0)	n = 4, 73.4 (56.0; 98.4)	0.07	(0.018 to 0.30)	<0.001	66.90
Consolidation y/n (%)	n = 3, 40.0 (4.0; 74.2)	n = 3, 0.0 (0.0; 16.0)	14.34	(0.59 to 347)	0.101	78.48
Reticulation (y/n) (%)	n = 2, 55.0 (10.0; 100.0)	n = 2, 74.0 (48.0; 100.0)	*0.12	(0.013 to 1.09)	0.06	0.00
Predominant GGO: GGO>(R+H) (%)	n = 2, 21.7 (14.8; 28.6)	n = 2, 7.1 (4.8; 9.4)	3.68	(1.22 to 11.09)	0.021	0.00
Predominant reticulation: (R+H)>GGO (%)	n = 2, 37.4 (28.6; 46.3)	n = 2, 74.6 (56.3; 92.9)	0.15	(0.032 to 0.67)	0.014	67.28
H and/or R > G (%)	n = 2, 2.0 (0.0; 4.0)	n = 2, 27.6 (14.3; 40.9)	0.06	(0.01 to 0.33)	0.001	0.00
Subpleural sparing (y/n) (%)	n = 3, 40.7 (30.8; 64.0)	n = 3, 4.3 (0.0; 9.3)	16.33	(2.28 to 117)	0.005	67.36
Peripheral predominance (outer 1/3) (%)	n = 6, 41.8 (33.3; 76.0)	n = 6, 83.3 (75.0; 95.5)	0.21	(0.11 to 0.38)	<0.001	16.22
Central predominance/perbronchial (inner 2/3) (%)	n = 5, 41.0 (0.0; 61.1)	n = 5, 8.0 (4.5; 14.7)	6.19	(2.4 to 15.7)	<0.001	34.29
Diffuse axial distribution both (%)	n = 5, 15.4 (5.6; 28.0)	n = 5, 4.5 (0.0; 21.7)	2.34	(1.07 to 5.11)	0.033	0.00
Upper lung predominant distribution (%)	n = 3, 0.0 (0.0; 2.8)	n = 3, 0.0 (0.0; 4.3)	0.53	(0.053 to 5.34)	0.592	0.00
Lower lung predominant (below carina) (%)	n = 4, 91.3 (80.0; 96.0)	n = 4, 92.0 (82.6; 100.0)	0.96	(0.27 to 3.36)	0.955	6.13
Diffuse distribution: both upper and lower lungs (%)	n = 3, 8.0 (6.3; 8.3)	n = 3, 0.0 (0.0; 17.4)	0.91	(0.23 to 3.63)	0.892	0.00
Continuous data	NSIP	UIP	Pooled MD	95%-CI	p-value	I-squared (%)
Age	n = 6, 54.8 (50.0; 59.2)	n = 6, 59.7 (53.1; 62.9)	-4.37	(-6.97 to -1.77)	0.001	30.90
TOT disease extent (% of lung volume)	n = 6, 27.9 (0.4; 37.1)	n = 6, 29.8 (0.2; 44.0)	-2.11	(-4.59 to 0.37)	0.095	0.00
GGO extent (% of Lung volume)	n = 7, 19.8 (5.6; 31.3)	n = 7, 17.6 (1.4; 20.0)	3.03	(1.31 to 4.75)	0.001	31.32
Honeycombing extent (% of lung volume)	n = 6, 1.1 (0.2; 12.5)	n = 6, 5.8 (1.5; 30.3)	-3.86	(-6.65 to -1.06)	0.007	90.45
Consolidation extent (% of lung volume)	n = 7, 3.0 (0.0; 10.8)	n = 7, 1.1 (0.0; 6.0)	1.79	(0.61 to 2.96)	0.003	58.88
Reticulation extent (% of lung volume)	n = 2, 8.2 (7.3; 9.0)	n = 2, 8.2 (6.8; 9.5)	-0.28	(-2.12 to 1.56)	0.763	0.00

GGO: ground-glass opacity, H: honeycombing; R: reticulation; y/n: yes/no; OR: odds ratio; CI: confidence interval; TOT: total; MD: mean difference

* One study was excluded because there were zero cells in both groups (all patients had the characteristic of interest)

**Table 4:** Random-effects meta-analysis according to DerSimonian and Laird and I^2.

	n studies	DerSimonian-Laird	95%-CI	p-value	I-squared	95%-CI
		Pooled OR				
*male*	6	0.32	(0.17 to 0.60)	<0.001	48.8%	(0.0 to 79.7)
*Smoking (ever and past-smokers)*	4	0.42	(0.23 to 0.76)	0.005	35.4%	(0.0 to 77.5)
*GGO (y/n)*	3 §	32.05	(0.08 to 12654.94)	0.256	92.4%	**
*Honeycombing (y/n)*	4	0.07	(0.02 to 0.30)	<0.001	66.9%	(0.0 to 88.2)
*Consolidation y/n*	3	14.34	(0.59 to 346.96)	0.101	78.5%	(16.5 to 92.4)
*Reticulation (y/n)*	2 §	0.12*	(0.01 to 1.10)	0.060	n.a.	**
*predominant GGO: GGO>(R+H)*	2	3.68	(1.22 to 11.09)	0.021	0.0%	**
*predominant reticulation: (R+H)>GGO*	2	0.15	(0.03 to 0.68)	0.014	67.3%	**
*H: H and/or R > G*	2	0.06	(0.01 to 0.32)	0.001	0.0%	**
*Relative subpleural spearing (y/n)*	3	16.33	(2.29 to 116.56)	0.005	67.4%	(0.0 to 89.3)
*peripheral predominance (outer 1/3)*	6	0.21	(0.11 to 0.39)	<0.001	16.2%	(0.0 to 78.7)
*central predominance/perbronchial (inner 2/3)*	5	6.19	(2.43 to 15.75)	<0.001	34.3%	(0.0 to 75.2)
*both inner outer*	5	2.34	(1.07 to 5.11)	0.033	0.0%	(0.0 to 79.2)
*upper predominant distribution*	3 §	0.53	(0.05 to 5.34)	0.592	0.0%	**
*lower (below carina/)*	4	0.96	(0.28 to 3.36)	0.955	6.1%	(0.0 to 85.6)
*both upper lower*	3	0.91	(0.23 to 3.63)	0.892	0.0%	(0.0 to 89.6)
		Pooled WMD				
*Age mean estimation*	6	-4.37	(-6.98 to -1.77)	0.001	30.9%	(0.0 to 73.5)
*TOT disease extent (mean % of Lung volume)*	6	-2.11	(-4.59 to 0.37)	0.095	0.0%	(0.0 to 84.7)
*GGO extent (mean % of Lung volume)*	7	3.03	(1.31 to 4.75)	0.001	31.3%	(0.0 to 73.7)
*Honeycombing extent (mean % of Lung volume)*	6	-3.86	(-6.65 to -1.06)	0.007	90.4%	(78.6 to 95.7)
*Consolidation extent (mean % of Lung volume)*	7	1.79	(0.62 to 2.96)	0.003	58.9%	(0.0 to 84.7)
*Reticulation extent (mean % of Lung volume)*	2	-0.28	(-2.13 to 1.56)	0.763	0.0%	**
*Emphysema extent (mean % of Lung volume)*	2	-1.40	(-4.97 to 2.16)	0.440	71.5%	**

* Single-study effect because one of the two studies were was excluded.

**No estimation possible because of, not enough too few studies.

In patients with NSIP, the extent of the pattern (% of the total lung volume, Table 2) was significantly less for honeycombing (NSIP: median 1.1%, UIP: 5.8%; MD -3.9; p = 0.007) and significantly greater for GGOs (NSIP: median 19.8%, UIP: 17.6%; MD 3.0; p = 0.007).

In patients with NSIP, the median central or peribronchovascular disease predominance (axial inner 2/3 of the lungs) was 41%, while in patients UIP, the median was only 8% (OR 6.2; p≤0.001). Additionally, a diffuse distribution with equal involvement of both inner and outer regions of the lungs was more often observed in patients with NSIP than in patients with UIP (median 15.4% vs. 4.5%; OR 2.3; p = 0.033). Along the z-axis, the median upper lung predominance was zero in both groups, whereas the median lower lobe predominance was greater than 90%.

### Results of individual studies

The individual results from each included study are summarized in forest plots for the most important variables that exhibited the best classification capabilities (Figs 4–12).

**Fig 4:**
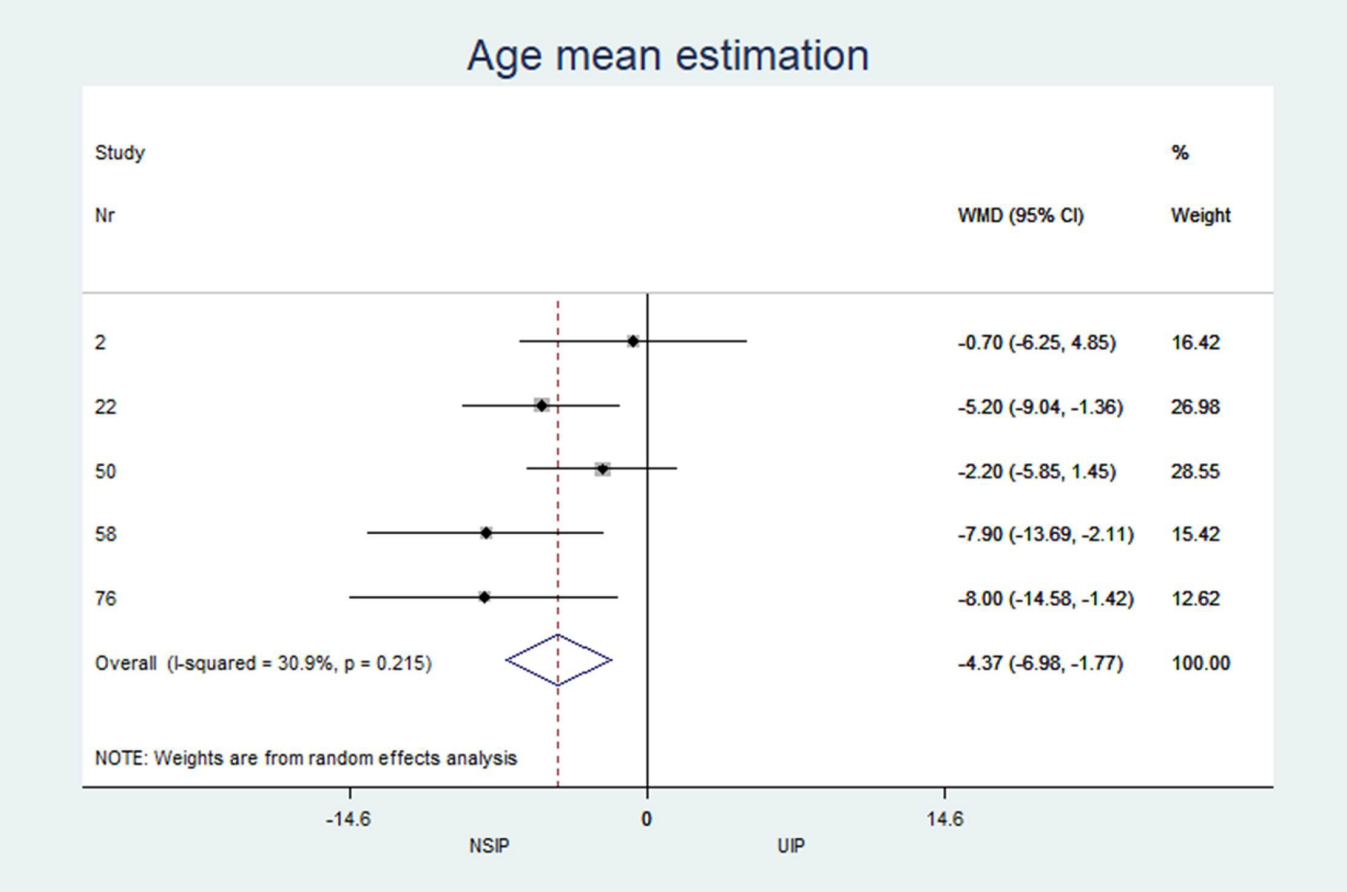
Mean age estimation.

**Fig 5:**
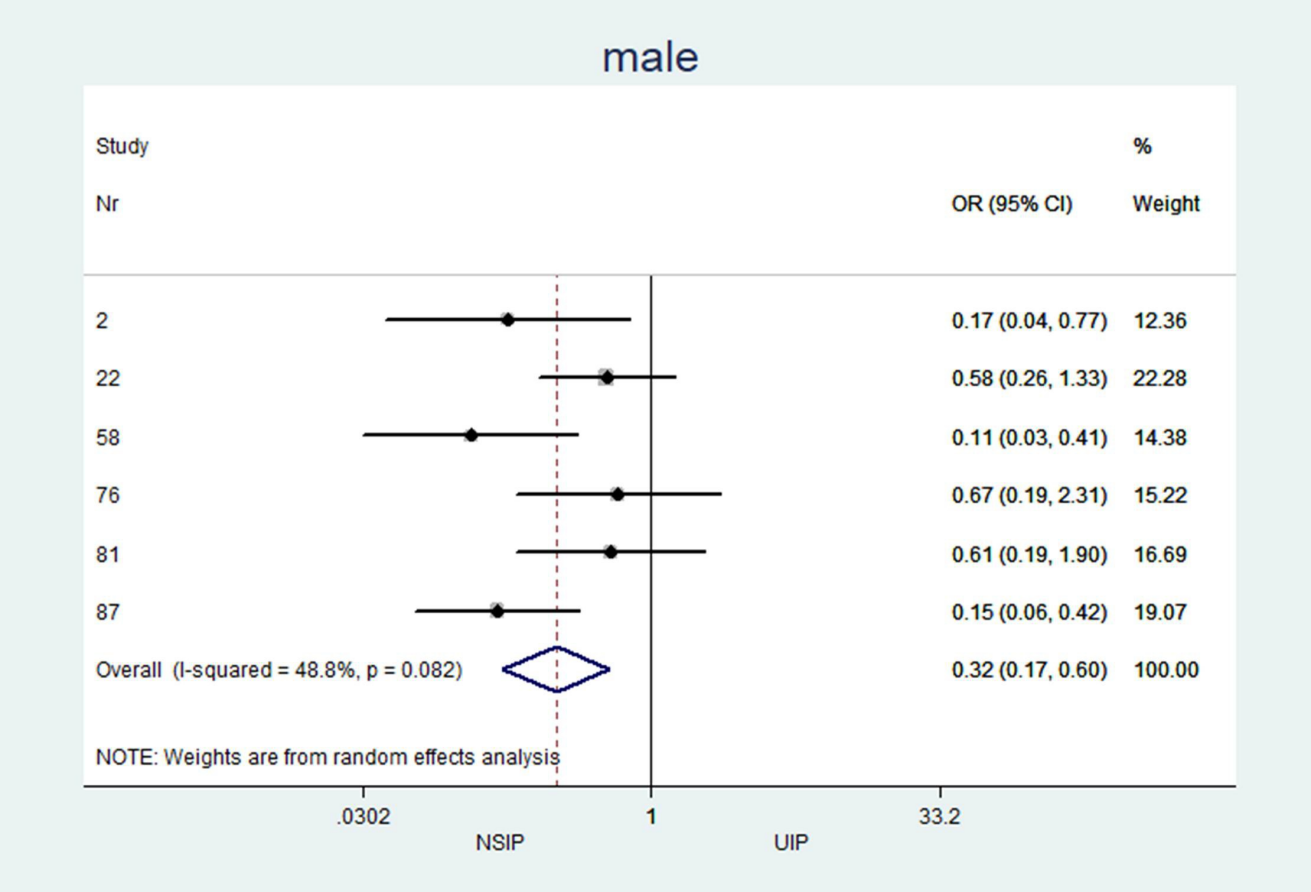
Male distribution.

**Fig 6:**
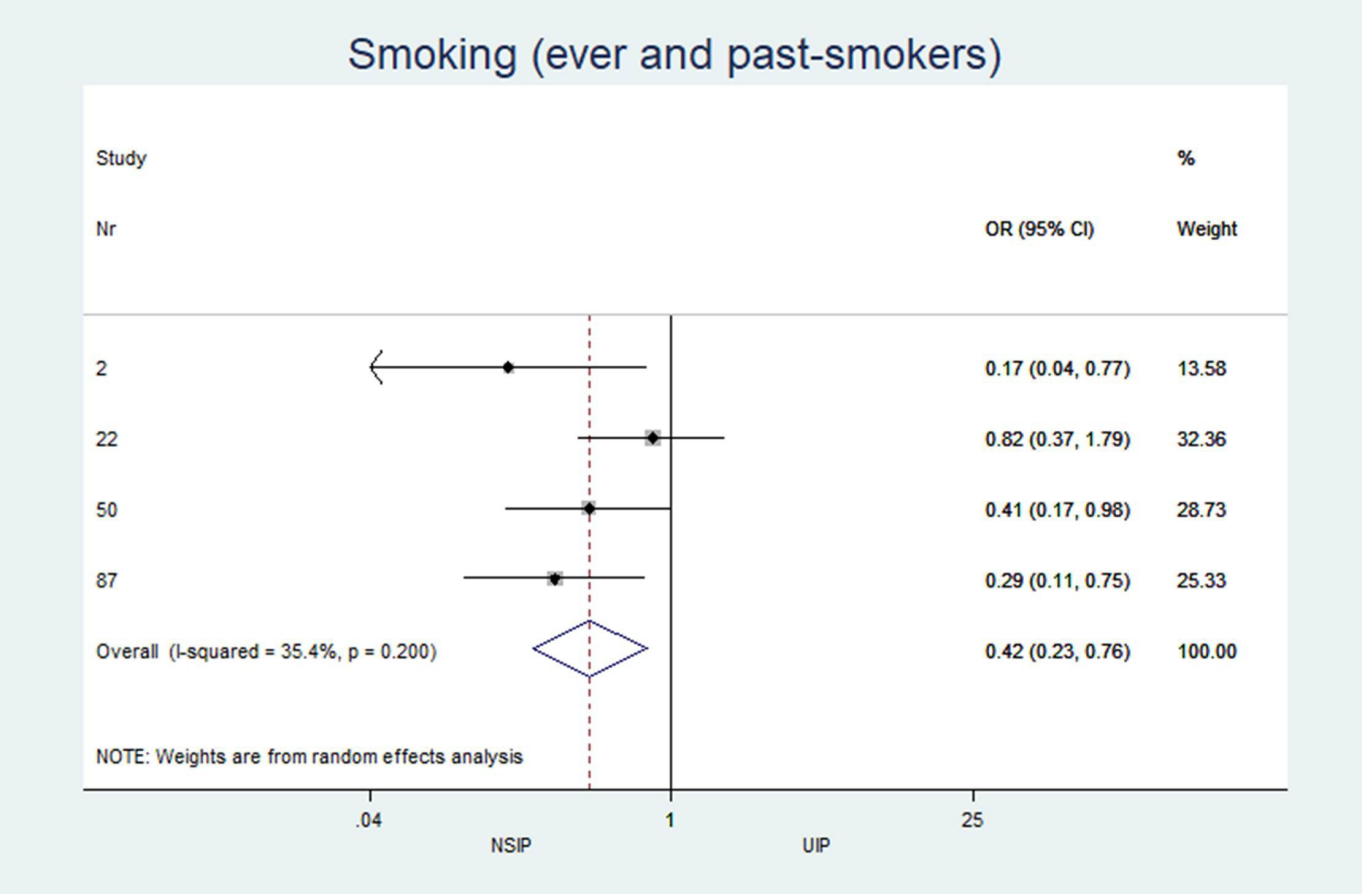
Smoking status.

**Fig 7:**
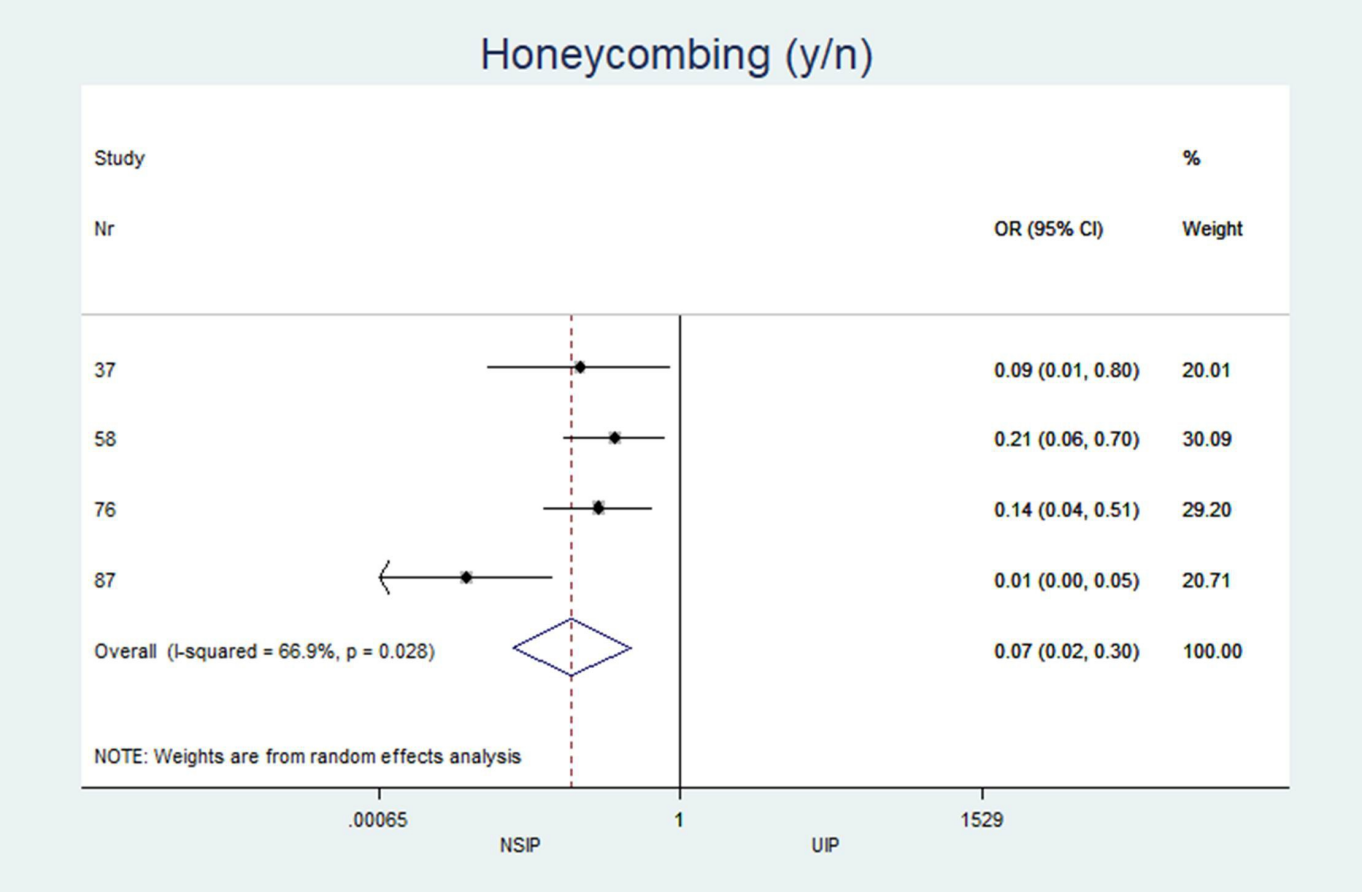
Presence of honey combing pattern.

**Fig 8:**
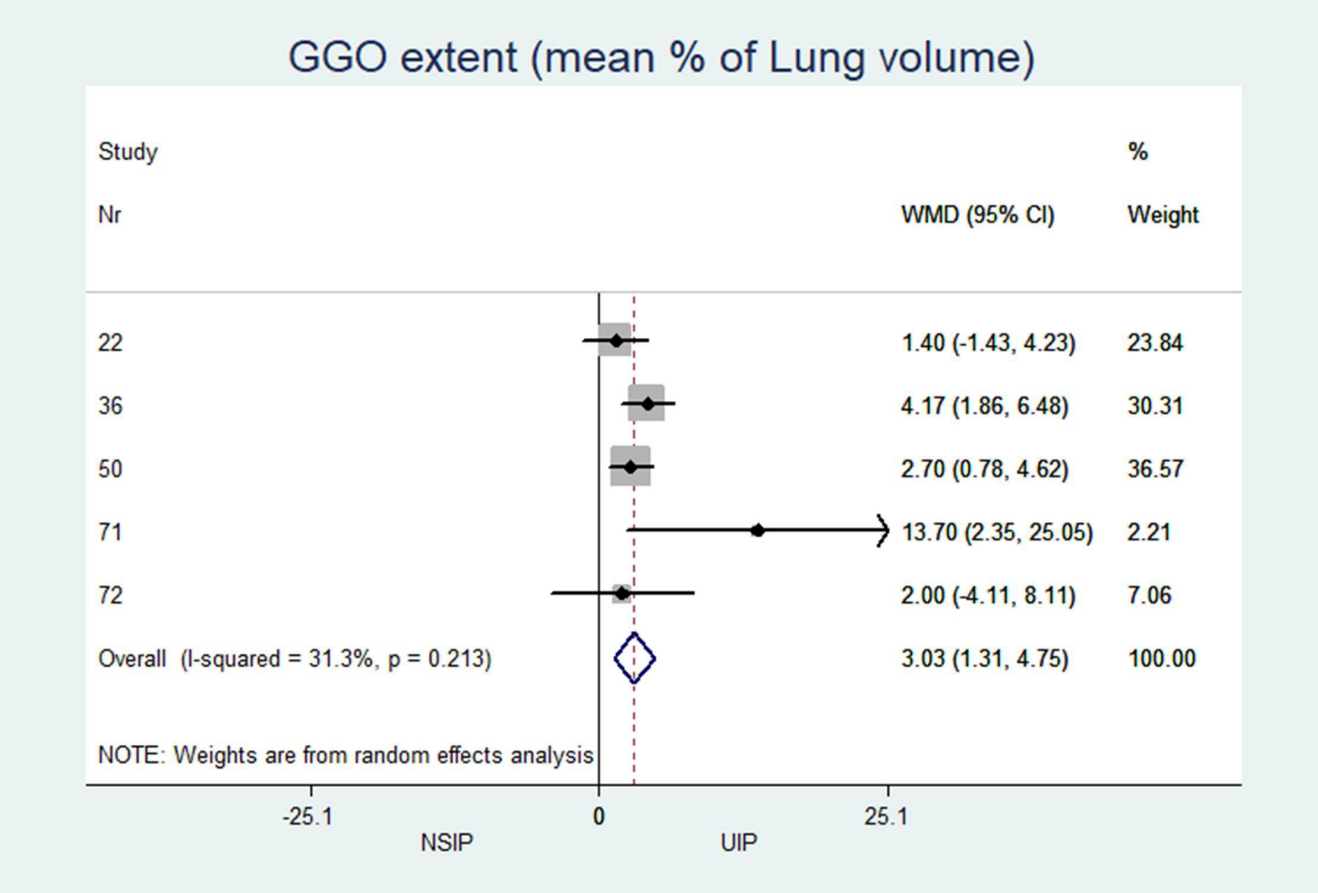
Extend of ground glas attenuation pattern in percent of total lung parenchyma.

**Fig 9:**
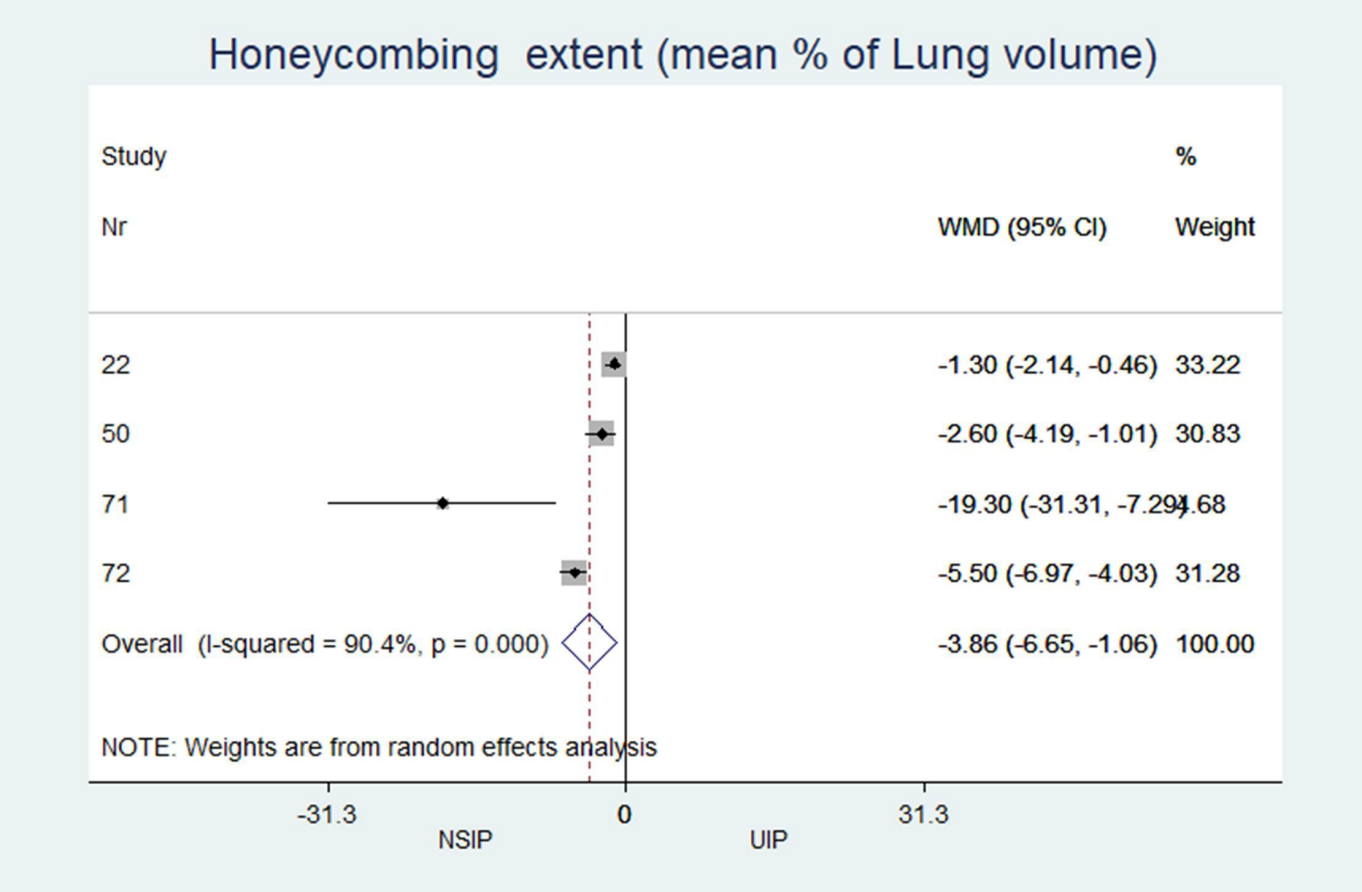
Extend of honey combing in percent of total lung parenchyma.

**Fig 10:**
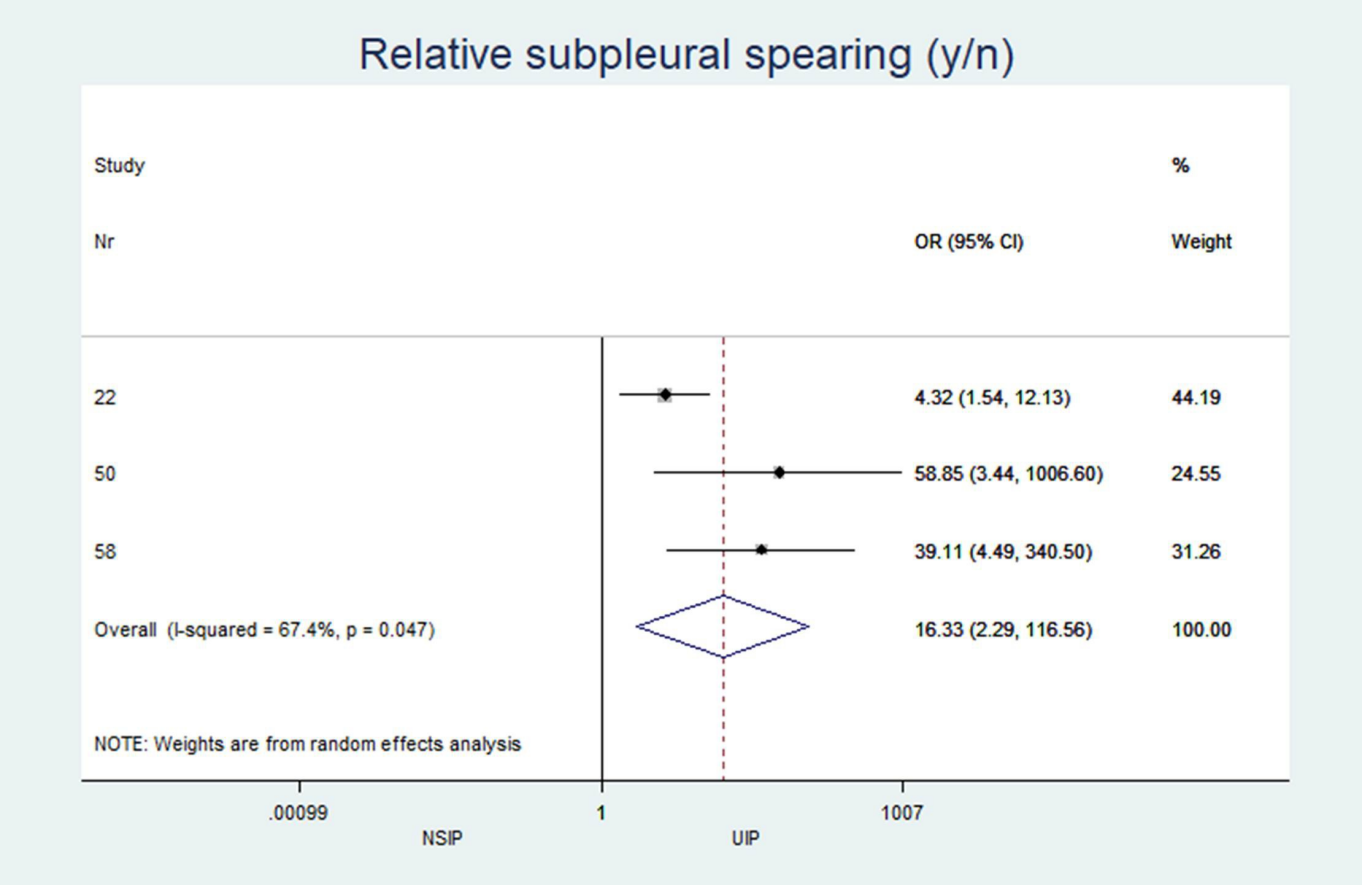
Presence of subpleural sparing.

**Fig 11:**
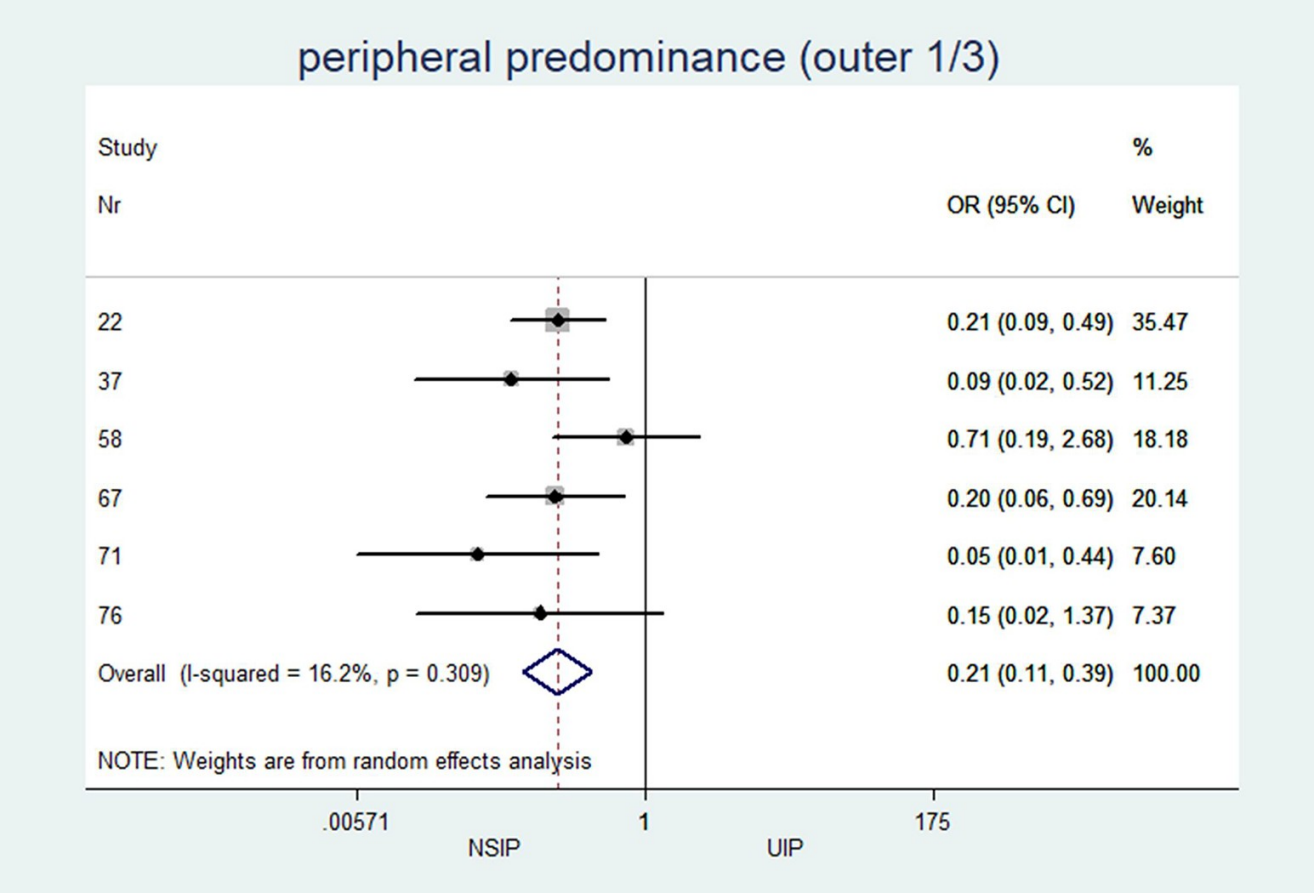
Peripheral predominance of CT patterns.

**Fig 12:**
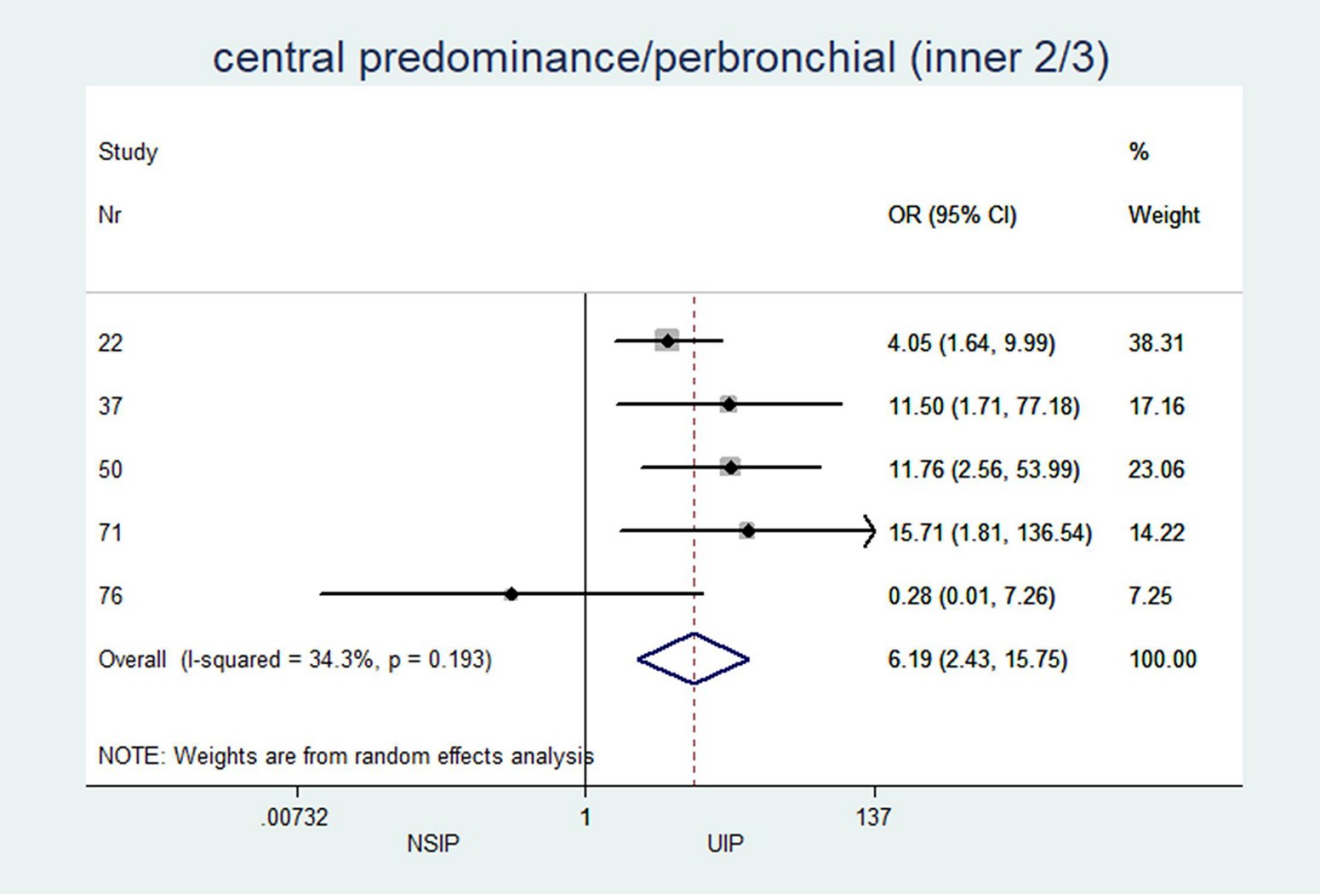
Central, peribronchovascular predominance of CT patterns.

### Heterogeneity (I^2^) of two-arm studies

The studies included in this meta-analysis displayed low heterogeneity in terms of clinical characteristics: age, sex and smoking habits had I^2^ values of 31%, 49% and 35%, respectively. The heterogeneity of the disease distribution was generally low (<35%), except for subpleural sparing, for which the I^2^ was moderate at 67%. The largest heterogeneity was observed in the pattern identified by the radiologists: GGOs showed severe heterogeneity (I^2^ = 92%), and honeycombing and consolidation displayed moderate heterogeneity (I^2^ = 67% and 78%, respectively).

## Discussion

The present results confirm that patient demographics, CT patterns and pattern distributions exhibited significant differences in patients with UIP and NSIP. The main discriminating factors were the presence of honeycombing, extent of GGOs, axial distribution, sex and age. Additionally, subpleural sparing and consolidation were mainly observed in patients with NSIP. Although these findings reflect the current diagnostic criteria [18,19]), to date, a comprehensive or formal meta-analysis of the published data has not been conducted. In this review of published data, we applied rigid inclusion criteria that relied on the aforementioned consensus statement. When applying the recommendations of the ATS, the European Respiratory Society (ERS) and Fleischner Society [11,18,19], only a few studies upheld this standard. For instance, an appropriate diagnosis of ILDs, mainly UIP, requires a multidisciplinary approach that takes into account clinical, radiological and pathological findings. By applying an interdisciplinary approach, the diagnostic accuracy and consequently the timely treatment of patients can be substantially improved. However, of more than 600 articles, only 33 studies adhered strictly to the proposed workflow and criteria for the management of these two specific patient populations. Additionally, the current recommendations regarding imaging patterns were determined by an expert panel.

Currently, the diagnostic algorithm is changing. Very recently, the Fleischner Society released a consensus paper introducing new diagnostic categories for the UIP pattern which has been adopted by the American Thoracic Society, European Respiratory Society, Japanese Respiratory Society, and Latin American Thoracic Society [35]. The former imaging categories were reviewed, and a new diagnostic category was introduced: CT pattern indeterminate for UIP. However, typical and probable UIP patterns are still considered categories with a very high probability of UIP, even though honeycombing might be absent in probable UIP cases. Although a new category has been introduced, histopathological confirmation of the categories of “CT pattern indeterminate for UIP” and “CT features most consistent with a non-IPF diagnosis” is still required. Although the diagnostic category of a “probable UIP pattern” does not include honeycombing on CT images, in the data presented in the current study, honeycombing appears to be the most reliable factor discriminating between UIP and NSIP patterns. This finding is most likely attributable to the weighting of honeycombing patterns among radiologists. In this meta-analysis, the pattern distribution did not prove to be particularly helpful for differentiating UIP and NSIP. Both entities showed a more basal and peripheral predominance. Additionally, subpleural sparing was an inconsistent finding in patients with NSIP. An important factor for differentiating UIP and NSIP is age and smoking history. IPF peaks at a significantly older age than NSIP [1–6]. This finding is supported by the data retrieved from the present analysis. This result supports the need for multidisciplinary ILD boards to incorporate a broad spectrum of clinical factors and to reach a final diagnosis. In summary, the present data not only shows honeycombing to be the most significant discriminator between UIP and NSIP but also the importance of clinical factors. Based on these results, we encourage radiologists to incorporate age, sex and smoking history into their diagnostic routine. Conversely, referring clinicians can benefit the most from the radiological reports when asking the reading radiologist to incorporate critical clinical data in the interpretation.

Currently, computer aided detection (CAD) systems based on artificial intelligence have become major topics of discussion in diagnostic radiology [36–45]. In previous studies using convolutional neural networks, computerized detection of CT patterns became feasible [46–48]. By combining automated CT pattern recognition algorithms and clinical and demographic characteristics of patients, the diagnosis of ILDs and, in particular, the differentiation of UIP patterns and NSIP patterns by machine learning algorithms could be feasible. The present meta-analysis of the imaging features of UIP and NSIP will also provide a necessary foundation for the further development of these algorithms, ultimately improving the diagnosis of ILDs and patient care. For instance, the ORs might be included in a Bayesian model, which would provide a probability-based diagnostic approach for UIP.

### Limitations

Our analysis has several limitations. First, the total number of studies and patients included was small. Although numerous studies have been published in the field, only a few studies fit our rigid inclusion criteria. Publication bias could not be assessed because we only had 2 to 7 studies in each meta-analysis. Another limitation might be the reference standards; some patient populations were confirmed by biopsy and some were diagnosed by imaging and ILD board consensus alone. Although these reference standards are consistent with clinical guidelines, this heterogeneity in the selection criteria might be questioned, presenting an incorporation bias. Some variables showed severe heterogeneity, probably due to the known interreader variability in CT patterns and the use of slightly different reference standards for cases that were diagnosed by an ILD board or histology. The different level of experience of the radiologists in each study may have influenced the results too: in the available method descriptions the median experience in chest imaging of the radiologists involved was 15.9 years, ranging from 10 to 22 years. The UIP and NSIP patients were analyzed by the same radiologists in all two arm studies, which may have helped counteracting this effect. Furthermore, the fact that some studies included secondary forms of fibrosis while others adhered to the idiopathic forms may have confounded the results of the study, although this hypothesis was not statistically confirmed.

The included studies did not investigate end-stage lungs affected by NSIP. In patients with terminal NSIP, honeycombing is almost always present, making discrimination from UIP nearly impossible. However, in end-stage lung disease, a radiological diagnosis and clinical options are very limited.

### Conclusions

In conclusion, this meta-analysis provides an overview of the main clinical features and CT patterns that discriminate between UIP and NSIP. Specifically, the honeycombing pattern is still the most specific factor discriminating between UIP and NSIP.

## Supporting information

S1 File PRISMA checklist. (DOC)

S2 File Embase search algorithm. (DOCX)

S3 File Comparison of the DerSimonian & Laird and Paule-Mandel approach. (DOCX)

## Abbreviations

UIP: Usual Interstitial Pneumonia
NSIP: Nonspecific Interstitial Pneumonia
ILD: Interstitial Lung Diseases
IPF: Idiopathic Pulmonary Fibrosis
IIP: Idiopathic Interstitial Pneumonia
HRCT: High Resolution Computed Tomography
CT: Computed Tomography
GGO: Ground Glass Opacity
MD: Mean Difference
OR: Odds Ratio
