# Supplementary material for: Meta-analysis of the radiological and clinical features of Usual Interstitial Pneumonia (UIP) and Nonspecific Interstitial Pneumonia (NSIP)
Source: PLoS One. 2020 Jan 13;15(1):e0226084. doi: 10.1371/journal.pone.0226084 (PMC6957301; doi:10.1371/journal.pone.0226084)
Supplement: S2 File — (DOCX) [file pone.0226084.s002.docx]

**S 2.** Embase search algorithm.

1 exp interstitial lung disease/di, rt [Diagnosis, Radiotherapy]

2 exp lung fibrosis/di, rt [Diagnosis, Radiotherapy]

3 1 or 2

4 exp radiography/

5 (pattern* or reticula* or honeycombing or ground-glass or peribronchovascular or bronchovascular or traction bronchiectasis or tractionbronchiectasis or UIP or NSIP).ti,ab,kw.

6 3 and 4 and 5

7 (fibro* or idiopathic* or pneumonia*).ti.

8 6 and 7

9 case report*.ti,ab.

10 8 not 9

11(therap* or treat*).ti.

12 10 not 11

13 limit 12 to yr="1992 -Current"
