# Supplementary material for: Meta-analysis of the radiological and clinical features of Usual Interstitial Pneumonia (UIP) and Nonspecific Interstitial Pneumonia (NSIP)
Source: PLoS One. 2020 Jan 13;15(1):e0226084. doi: 10.1371/journal.pone.0226084 (PMC6957301; doi:10.1371/journal.pone.0226084)
Supplement: S3 File — (DOCX) [file pone.0226084.s003.docx]

| \| **S 3:** Comparison of the DerSimonian & Laird and Paule-Mandel approach.   \|  \| n studies \| DerSimonian-Laird \| 95%-CI \| p-value \| I-squared \| 95%-CI \| Paule-Mandel \| 95%-CI \| p-value \| I-squared \| \| --- \| --- \| --- \| --- \| --- \| --- \| --- \| --- \| --- \| --- \| --- \| \|  \|  \| Pooled OR \|  \|  \|  \|  \| Pooled OR \|  \|  \|  \| \| male \| 6 \| 0.32 \| (0.17 to 0.60) \| <0.001 \| 48.8 % \| (0.0 to 79.7) \| 0.32 \| (0.17 to 0.60) \| <0.001 \| 48.2 % \| \| Smoking (ever and past-smokers) \| 4 \| 0.42 \| (0.23 to 0.76) \| 0.005 \| 35.4 % \| (0.0 to 77.5) \| 0.42 \| (0.23 to 0.76) \| 0.005 \| 34.8 % \| \| GGO (y/n) \| 3 § \| 32.05 \| (0.08 to 12654.94) \| 0.256 \| 92.4 % \| ** \| 31.42 \| (0.12 to 8144.19) \| 0.224 \| 91.3 % \| \| Honeycombing (y/n) \| 4 \| 0.07 \| (0.02 to 0.30) \| <0.001 \| 66.9 % \| (0.0 to 88.2) \| 0.07 \| (0.02 to 0.33) \| 0.001 \| 71.8 % \| \| Consolidation y/n \| 3 \| 14.34 \| (0.59 to 346.96) \| 0.101 \| 78.5 % \| (16.5 to 92.4) \| 14.22 \| (0.68 to 295.75) \| 0.086 \| 76.3 % \| \| Reticulation (y/n) \| 2 § \| 0.12* \| (0.01 to 1.10) \| 0.060 \| n.a. \| ** \| n.a. \| n.a. \| n.a. \| n.a. \| \| predominant GGO: GGO>(R+H) \| 2 \| 3.68 \| (1.22 to 11.09) \| 0.021 \| 0.0 % \| ** \| n.a. \| n.a. \| n.a. \| n.a. \| \| predominant reticulation: (R+H)>GGO \| 2 \| 0.15 \| (0.03 to 0.68) \| 0.014 \| 67.3 % \| ** \| n.a. \| n.a. \| n.a. \| n.a. \| \| H: H and/or R > G \| 2 \| 0.06 \| (0.01 to 0.32) \| 0.001 \| 0.0 % \| ** \| n.a. \| n.a. \| n.a. \| n.a. \| \| Relative subpleural spearing (y/n) \| 3 \| 16.33 \| (2.29 to 116.56) \| 0.005 \| 67.4 % \| (0.0 to 89.3) \| 14.64 \| (2.79 to 76.89) \| 0.002 \| 55.2 % \| \| peripheral predominance (outer 1/3) \| 6 \| 0.21 \| (0.11 to 0.39) \| <0.001 \| 16.2 % \| (0.0 to 78.7) \| 0.21 \| (0.11 to 0.39) \| <0.001 \| 18.6 % \| \| central predominance/perbronchial (inner 2/3) \| 5 \| 6.19 \| (2.43 to 15.75) \| <0.001 \| 34.3 % \| (0.0 to 75.2) \| 6.18 \| (2.11 to 18.10) \| 0.001 \| 48.3 % \| \| both inner outer \| 5 \| 2.34 \| (1.07 to 5.11) \| 0.033 \| 0.0 % \| (0.0 to 79.2) \| 2.34 \| (1.07 to 5.11) \| 0.033 \| 0.0 % \| \| upper predominant distribution \| 3 § \| 0.53 \| (0.05 to 5.34) \| 0.592 \| 0.0 % \| ** \| 0.53 \| (0.05 to 5.34) \| 0.592 \| 0.0 % \| \| lower (below carina/) \| 4 \| 0.96 \| (0.28 to 3.36) \| 0.955 \| 6.1 % \| (0.0 to 85.6) \| 0.96 \| (0.28 to 3.36) \| 0.955 \| 6.0 % \| \| both upper lower \| 3 \| 0.91 \| (0.23 to 3.63) \| 0.892 \| 0.0 % \| (0.0 to 89.6) \| 0.91 \| (0.23 to 3.63) \| 0.892 \| 0.0 % \| \|  \|  \| Pooled MD \|  \|  \|  \|  \| Pooled MD \|  \|  \|  \| \| Age mean estimation \| 6 \| -4.37 \| (-6.98 to -1.77) \| 0.001 \| 30.9 % \| (0.0 to 73.5) \| -4.39 \| (-7.06 to -1.73) \| 0.001 \| 33.9 % \| \| TOT disease extent (mean % of Lung volume) \| 6 \| -2.11 \| (-4.59 to 0.37) \| 0.095 \| 0.0 % \| (0.0 to 84.7) \| -2.11 \| (-4.59 to 0.37) \| 0.095 \| 0.0 % \| \| GGO extent (mean % of Lung volume) \| 7 \| 3.03 \| (1.31 to 4.75) \| 0.001 \| 31.3 % \| (0.0 to 73.7) \| 3.12 \| (0.87 to 5.37) \| 0.006 \| 55.7 % \| \| Honeycombing extent (mean % of Lung volume) \| 6 \| -3.86 \| (-6.65 to -1.06) \| 0.007 \| 90.4 % \| (78.6 to 95.7) \| -5.58 \| (-12.39 to 1.23) \| 0.109 \| 98.5 % \| \| Consolidation extent (mean % of Lung volume) \| 7 \| 1.79 \| (0.62 to 2.96) \| 0.003 \| 58.9 % \| (0.0 to 84.7) \| 2.10 \| (0.19 to 4.01) \| 0.031 \| 84.5 % \| \| Reticulation extent (mean % of Lung volume) \| 2 \| -0.28 \| (-2.13 to 1.56) \| 0.763 \| 0.0 % \| ** \| n.a. \| n.a. \| n.a. \| n.a. \| \| Emphysema extent (mean % of Lung volume) \| 2 \| -1.40 \| (-4.97 to 2.16) \| 0.440 \| 71.5 % \| ** \| n.a. \| n.a. \| n.a. \| n.a. \| \| \| --- \| --- \| --- \| --- \| --- \| --- \| --- \| --- \| --- \| --- \| --- \| --- \| --- \| --- \| --- \| --- \| --- \| --- \| --- \| --- \| --- \| --- \| --- \| --- \| --- \| --- \| --- \| --- \| --- \| --- \| --- \| --- \| --- \| --- \| --- \| --- \| --- \| --- \| --- \| --- \| --- \| --- \| --- \| --- \| --- \| --- \| --- \| --- \| --- \| --- \| --- \| --- \| --- \| --- \| --- \| --- \| --- \| --- \| --- \| --- \| --- \| --- \| --- \| --- \| --- \| --- \| --- \| --- \| --- \| --- \| --- \| --- \| --- \| --- \| --- \| --- \| --- \| --- \| --- \| --- \| --- \| --- \| --- \| --- \| --- \| --- \| --- \| --- \| --- \| --- \| --- \| --- \| --- \| --- \| --- \| --- \| --- \| --- \| --- \| --- \| --- \| --- \| --- \| --- \| --- \| --- \| --- \| --- \| --- \| --- \| --- \| --- \| --- \| --- \| --- \| --- \| --- \| --- \| --- \| --- \| --- \| --- \| --- \| --- \| --- \| --- \| --- \| --- \| --- \| --- \| --- \| --- \| --- \| --- \| --- \| --- \| --- \| --- \| --- \| --- \| --- \| --- \| --- \| --- \| --- \| --- \| --- \| --- \| --- \| --- \| --- \| --- \| --- \| --- \| --- \| --- \| --- \| --- \| --- \| --- \| --- \| --- \| --- \| --- \| --- \| --- \| --- \| --- \| --- \| --- \| --- \| --- \| --- \| --- \| --- \| --- \| --- \| --- \| --- \| --- \| --- \| --- \| --- \| --- \| --- \| --- \| --- \| --- \| --- \| --- \| --- \| --- \| --- \| --- \| --- \| --- \| --- \| --- \| --- \| --- \| --- \| --- \| --- \| --- \| --- \| --- \| --- \| --- \| --- \| --- \| --- \| --- \| --- \| --- \| --- \| --- \| --- \| --- \| --- \| --- \| --- \| --- \| --- \| --- \| --- \| --- \| --- \| --- \| --- \| --- \| --- \| --- \| --- \| --- \| --- \| --- \| --- \| --- \| --- \| --- \| --- \| --- \| --- \| --- \| --- \| --- \| --- \| --- \| --- \| --- \| --- \| --- \| --- \| --- \| --- \| --- \| --- \| --- \| --- \| --- \| --- \| --- \| --- \| --- \| --- \| --- \| --- \| --- \| --- \| --- \| --- \| --- \| --- \| --- \| --- \| --- \| --- \| --- \| --- \| --- \| --- \| --- \| --- \| --- \| --- \| --- \| --- \| |
| --- | --- | --- | --- | --- | --- | --- | --- | --- | --- | --- | --- | --- | --- | --- | --- | --- | --- | --- | --- | --- | --- | --- | --- | --- | --- | --- | --- | --- | --- | --- | --- | --- | --- | --- | --- | --- | --- | --- | --- | --- | --- | --- | --- | --- | --- | --- | --- | --- | --- | --- | --- | --- | --- | --- | --- | --- | --- | --- | --- | --- | --- | --- | --- | --- | --- | --- | --- | --- | --- | --- | --- | --- | --- | --- | --- | --- | --- | --- | --- | --- | --- | --- | --- | --- | --- | --- | --- | --- | --- | --- | --- | --- | --- | --- | --- | --- | --- | --- | --- | --- | --- | --- | --- | --- | --- | --- | --- | --- | --- | --- | --- | --- | --- | --- | --- | --- | --- | --- | --- | --- | --- | --- | --- | --- | --- | --- | --- | --- | --- | --- | --- | --- | --- | --- | --- | --- | --- | --- | --- | --- | --- | --- | --- | --- | --- | --- | --- | --- | --- | --- | --- | --- | --- | --- | --- | --- | --- | --- | --- | --- | --- | --- | --- | --- | --- | --- | --- | --- | --- | --- | --- | --- | --- | --- | --- | --- | --- | --- | --- | --- | --- | --- | --- | --- | --- | --- | --- | --- | --- | --- | --- | --- | --- | --- | --- | --- | --- | --- | --- | --- | --- | --- | --- | --- | --- | --- | --- | --- | --- | --- | --- | --- | --- | --- | --- | --- | --- | --- | --- | --- | --- | --- | --- | --- | --- | --- | --- | --- | --- | --- | --- | --- | --- | --- | --- | --- | --- | --- | --- | --- | --- | --- | --- | --- | --- | --- | --- | --- | --- | --- | --- | --- | --- | --- | --- | --- | --- | --- | --- | --- | --- | --- | --- | --- | --- | --- | --- | --- | --- | --- | --- | --- | --- | --- | --- | --- | --- | --- | --- | --- | --- | --- | --- | --- | --- | --- | --- |
